# DNA damage and repair kinetics after microbeam radiation therapy emulation in living cells using monoenergetic synchrotron X-ray microbeams

**DOI:** 10.1107/S0909049511011836

**Published:** 2011-05-14

**Authors:** Carl N. Sprung, Marian Cholewa, Noriko Usami, Katsumi Kobayashi, Jeffrey C. Crosbie

**Affiliations:** a Centre for Women’s Health Research and Centre for Innate Immunology and Infectious Disease, Monash Institute for Medical Research, Monash University, Clayton, Victoria, Australia; b Monash Centre for Synchrotron Science, Monash University, Clayton, Victoria, Australia; c Photon Factory, KEK, Tsukuba, Japan; d William Buckland Radiotherapy Centre, Alfred Hospital, Melbourne, Victoria, Australia

**Keywords:** double-strand breaks, ionizing radiation, X-rays, γH2AX

## Abstract

A novel synchrotron-based approach, known as microbeam radiation therapy (MRT), currently shows considerable promise in increased tumour control and reduced normal tissue damage compared with conventional radiotherapy. Different microbeam widths and separations were investigated using a controlled cell culture system and monoenergetic (5.35 keV) synchrotron X-rays in order to gain further insight into the underlying cellular response to MRT. DNA damage and repair was measured using fluorescent antibodies against phosphorylated histone H2AX, which also allowed us to verify the exact location of the microbeam path. Beam dimensions that reproduced promising MRT strategies were used to identify useful methods to study the underpinnings of MRT. These studies include the investigation of different spatial configurations on bystander effects. γH2AX foci number were robustly induced in directly hit cells and considerable DNA double-strand break repair occurred by 12 h post-10 Gy irradiation; however, many cells had some γH2AX foci at the 12 h time point. γH2AX foci at later time points did not directly correspond with the targeted regions suggesting cell movement or bystander effects as a potential mechanism for MRT effectiveness. Partial irradiation of single nuclei was also investigated and in most cases γH2AX foci were not observed outside the field of irradiation within 1 h after irradiation indicating very little chromatin movement in this time frame. These studies contribute to the understanding of the fundamental radiation biology relating to the MRT response, a potential new therapy for cancer patients.

## Abstract 2

The molecular response of mammalian cells to a monoenergetic synchrotron X-ray microbeam which emulated microbeam radiation configurations has been investigated. Very few γH2AX foci were found outside the irradiated zone within 1 h of irradiation, even within a single nucleus. Furthermore, 12 h after radiation there was a large decrease in foci number but many cells still contained γH2AX foci, of which many were outside the directly irradiated regions.

## 1. Introduction

It is estimated that as many as 52% of all cancer patients should receive radiotherapy (RT) (Delaney *et al.*, 2005 ▶); therefore, investigations into advances in this modality are critical. Currently, conventional RT doses are limited by adverse effects to normal tissues. Therefore, a primary goal in radiation oncology is to find a RT strategy that would increase the therapeutic index (the ratio of curative effects to adverse normal tissue effects).

### 1.1. MRT

One such advance is microbeam radiation therapy (MRT), in which an array of high-dose planar X-ray microbeams (50–250 keV) are delivered to a tumour site. Huge photon fluxes are required to deliver effective amounts of dose on a scale of micrometres. Synchrotrons are capable of generating such high-dose-rate X-ray microbeams with physical properties that conventional sources do not possess. Investigations into synchrotron MRT in animal models have indicated that tumours can be ablated by MRT at radiation levels that spare normal tissues (Dilmanian *et al.*, 2002 ▶, 2003 ▶, 2005 ▶; Laissue *et al.*, 1998 ▶, 2007 ▶; Miura *et al.*, 2006 ▶; Smilowitz *et al.*, 2006 ▶; Regnard, Brauer-Krisch *et al.*, 2008 ▶; Regnard, Le Duc *et al.*, 2008 ▶; Serduc *et al.*, 2008 ▶; Crosbie *et al.*, 2010 ▶). Therefore, the therapeutic index for MRT has the promise of being greater than conventional radiotherapy and may improve radiation-based cancer treatment. The reason for this difference in response is presently unknown but a possible mechanism is inter-cellular effects between maximally and minimally irradiated cells. Indirect effects such as these are an important phenomenon and a candidate for the beneficial mechanism responsible for the observed therapeutic index increase in animal models following MRT (Crosbie *et al.*, 2010 ▶).

### 1.2. Bystander effect

In the past, the paradigm was that only the directly irradiated cells were affected by the radiation, but in recent years a paradigm shift has occurred that indicates cells outside the effects of direct irradiation respond to the radiation, including affecting the DNA (Nagasawa & Little, 1992 ▶; Littlefield *et al.*, 1969 ▶; Prise & O’Sullivan, 2009 ▶; Yang *et al.*, 2005 ▶; Han *et al.*, 2007 ▶; Seymour & Mothersill, 1997 ▶).

The bystander effect is a response that occurs in cells in close proximity to cells that have been directly exposed to radiation (Mothersill & Seymour, 1997 ▶; Seymour & Mothersill, 2006 ▶; Gerashchenko & Howell, 2003 ▶; Azzam *et al.*, 1998 ▶). Bystander effects have been shown to involve numerous cellular changes including micronucleus formation (Belyakov *et al.*, 2001 ▶), cell death (Mothersill & Seymour, 1997 ▶; Lyng *et al.*, 2000 ▶), effects on clonogenicity (Mothersill & Seymour, 1997 ▶; Iyer *et al.*, 2000 ▶), neoplastic transformation (Lewis *et al.*, 2001 ▶), increase in reactive oxygen species (Narayanan *et al.*, 1997 ▶; Azzam *et al.*, 2002 ▶), induction of chromosome aberrations (Littlefield *et al.*, 1969 ▶), sister chromosome exchanges, mutations (Nagasawa & Little, 1992 ▶), genomic instability (Kadhim *et al.*, 1992 ▶), DNA double-strand breaks (Han *et al.*, 2007 ▶; Yang *et al.*, 2005 ▶) and transcription alterations (Iyer *et al.*, 2000 ▶; Hickman *et al.*, 1994 ▶). The bystander effect has been shown to function through a number of mechanisms including *via* soluble factors, such as chemokines and cytokines, which can act over longer distances, or signalling *via* gap junctions, which occurs between adjacent cells (Hei *et al.*, 2008 ▶; Bentzen, 2006 ▶). In MRT, cells in the valley region between the microbeam peaks may receive radiation doses of several Gray. A bystander effect has been hypothesized to occur following MRT (Dilmanian *et al.*, 2005 ▶; Kashino *et al.*, 2009 ▶). However, we note a distinction between *in vivo* MRT (cell–cell communication between maximally and minimally irradiated cells) and the classical definition of the bystander effect, in which neighbouring cells receive zero dose. Bystander effects have been observed in human fibroblasts when individual cell nuclei were targeted using a monoenergenic X-ray microbeam (Tomita *et al.*, 2010 ▶).

### 1.3. DNA damage and repair

Ionizing radiation can cause DNA double-strand breaks, a critical type of lesion for which the cell has a number of mechanisms to deal with depending on lesion severity, complexity and cell sensitivity. Typically a cell can repair the double-strand break or undergo apoptosis. The number of DNA double-strand breaks can be accurately estimated since there is an almost one-to-one correlation to phosphorylation of histone H2AX. γH2AX foci form relatively soon after irradiation and the phosphorylation of H2AX spans a two megabase region around a DNA double-strand break which is observed as individual foci in the nucleus (Sedelnikova *et al.*, 2002 ▶).

Here, we investigate the effect of different spatial X-ray microbeam configurations using DNA double-strand breakage and repair as the endpoint. We found γH2AX foci were robustly induced in directly targeted cells and, although the presence of γH2AX foci was evident 12 h post-irradiation, many cells still had residual foci. At the later time points these foci did not directly correspond with the targeted regions. Partial irradiation of single nuclei was also investigated and in very few cases was a γH2AX focus observed within 1 h after irradiation outside the precise field of irradiation. These experiments are important for gaining an understanding of the cellular response to radiation damage which contributes to the knowledge base in an effort to improve radiotherapy regimes for cancer patients.

## 2. Methods

### 2.1. Cell lines

V79 Chinese hamster lung cells and NB1-RGB (human fibroblasts) cells were grown in DMEM, 10% FBS and antibiotics in a humidified 5% CO_2_ environment. Thirty minutes prior to irradiation, 33258 Hoerchst dye was added to enable visualization of chromatin for computer-assisted targeting. V79 cells were provided by Dr Kasai-Eguchi (Kanai *et al.*, 1997 ▶). NB1-RGB was obtained from the RIKEN Bio-resource Center Cell Bank (Tsukuba, Ibaraki, Japan).

### 2.2. Irradiation of cells

The cell irradiations were carried out on beamline BL27B at the Photon Factory synchrotron (part of the KEK high-energy accelerator research organization) in Tsukuba, Japan, using a 10 µm × 10 µm monoenergetic (5.35 keV) X-ray beam. We used specially designed cell dish jigs which allowed us to transfer positioning coordinates from the beamline microscope to an off-line confocal microscope. A more complete description of the system is given elsewhere (Usami *et al.*, 2006 ▶; Maeda *et al.*, 2008 ▶; Kobayashi *et al.*, 2001 ▶). Cells grown on Mylar membrane bottomed dishes were given 5 ml of fresh media, and a water immersion lens (×40) was used to identify cellular targets. The exposure rate was approximately 30 R s^−1^ which is calculated to be equivalent to a dose rate of approximately 0.3 Gy s^−1^. The dose rate was measured using an AXUV-100 absolute XUV silicon photodiode (International Radiation Detectors, Torrance, CA, USA) (Maeda *et al.*, 2008 ▶). The conversion of 30 R s^−1^ to approximately 0.3 Gy s^−1^ is derived from the relationship of exposure (R) to air kerma (dose in air) (Gy) and described by Greening (1985 ▶),
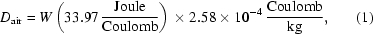
where *W* is the conversion factor from exposure to dose and is the energy required to create an ion pair in air (33.97 J C^−1^), and 1 R = 2.58 × 10^−4^ C kg^−1^. Therefore,
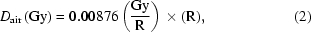
which is approximated to
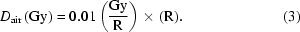
Hence 30 R s^−1^ ≃ 0.3 Gy s^−1^.

### 2.3. γH2AX immunofluorescence staining

After irradiation of regions with 2 Gy, 5 Gy or 10 Gy, the slides were fixed for 20 min with ice-cold 100% methanol and then a quick exposure to ice-cold acetic acid. The Mylar dishes were rinsed with three 5 min phosphate-buffered saline (PBS) washes. Slides were treated three times for 10 min in a blocking solution of 3% bovine serum albumin in PBS. Mouse anti-γH2AX antibody (Upstate, NY, USA) was added (1:500 in PBS) and incubated for 2 h in the dark at room temperature. The primary antibody was washed off with three PBS washes, then the plates were exposed to a secondary goat anti-mouse antibody (1:500 in PBS) conjugated with Alexa-488 (Molecular Probes, Oregon, USA) and incubated for 1 h in the dark at room temperature. Slides were rinsed three times in PBS and stained with 1 µg ml^−1^ propidium iodide. γH2AX images were acquired using an Olympus Fluoview confocal system adapted to an Olympus BX51W1 fluorescent microscope with a 40× water immersion objective (Olympus, Shinjuku-ku, Tokyo, Japan). Eight to ten *Z* sections with a 0.5 µm step size were de-convoluted to obtain γH2AX images.

## 3. Results

### 3.1. *In vitro* MRT configurations

**Figure 1:**
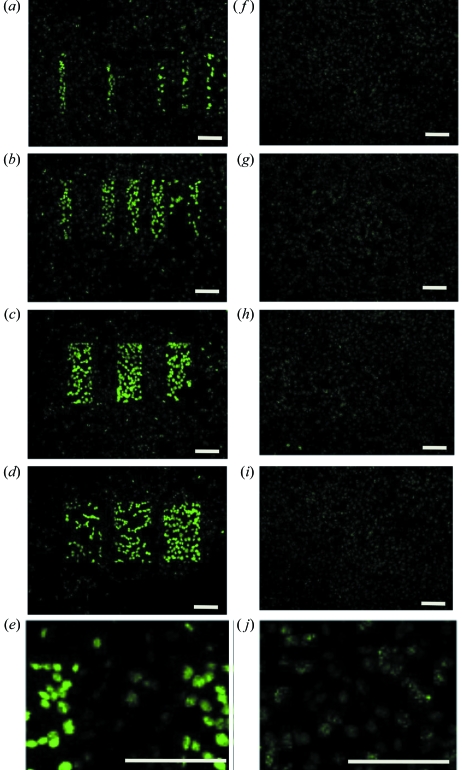
DNA double-strand break repair in V79 cells after MRT pattern emulation. Fluorescent staining with the γH2AX antibody (a marker of DNA damage) shows synchrotron MRT emulation in cell culture using a slit system of varying beam widths and interbeam spacings. The in-air entrance dose was 10 Gy in all cases and the cells (V79 Chinese hamster lung cells) were fixed 1 h (*a*–*e*) and 12 h (*f*–*j*) post-irradiation. (*a*, *f*) 25 µm beam width with centre-to-centre spacings of between 50 and 300 µm, (*b*, *g*) 50 µm beam width with spacings of between 75 and 300 µm, (*c*, *h*) 100 µm beam width with a 200 µm centre-to-centre spacing, and (*d*, *i*) 150 µm beam width and a 200 µm spacing. (*e*, *j*) Zoomed images from 100 µm beam width with a 200 µm centre-to-centre spacing. Bars indicate 100 µm.

We were able to test and clearly observe the exact targeting of the microbeam using fluorescently tagged γH2AX as a marker with separations consistent with MRT configurations previously used in animal studies. We selected a number of different configurations, including 25 µm-wide beams with centre-to-centre spacings of 50 µm, 75 µm, 100 µm, 175 µm, 200 µm and 300 µm; 50 µm-wide beams with centre-to-centre spacings of 75 µm, 125 µm, 225 µm and 325 µm; 100 µm-wide beams with centre-to-centre spacings of 200 µm; and 150 µm-wide beams with centre-to-centre spacings of 200 µm (Fig. 1 ▶).

### 3.2. Late responses to *in vitro* MRT

**Figure 2:**
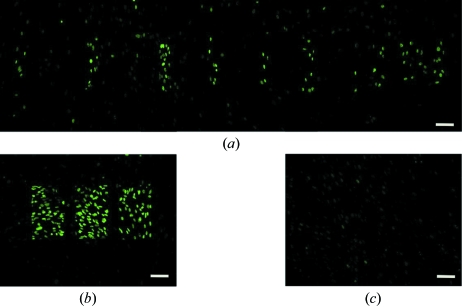
Fluorescent staining with the γH2AX antibody shows synchrotron MRT emulation in NB1-RGB human fibroblast cultured cells. The geometry of the microbeams was evident 1 h post-irradiation with (*a*) 50 µm beam width and spacings of between 75 and 300 µm and (*b*) 150 µm beam width with a 200 µm spacing. Most but not all of the DNA damage was repaired by 12 h post-irradiation as shown in a representative area of the 12 h 50 µm beam sample (*c*). The in-air entrance dose was 10 Gy in all cases. Bars indicate 100 µm.

We observed a large decrease in γH2AX staining intensity in the regions directly irradiated with the microbeam by 12 h post-irradiation. At this 12 h time point, individual foci within the nucleus were evident but they were spread across the region of MRT irradiation [Figs. 1(*f*)–1(*j*) ▶]. The lower intensity and dispersion of cells with foci made it difficult to observe the targeted patterning of X-rays as seen at the 1 h post-irradiation time point. Similar results were observed in a human fibroblast cell line (NB1-RGB; Fig. 2 ▶).

### 3.3. Dose response of MRT

**Figure 3:**
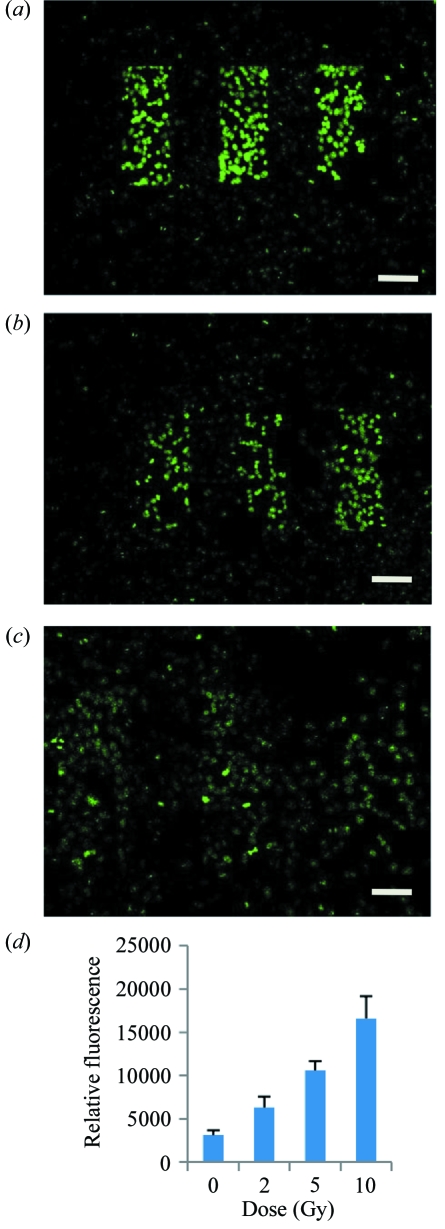
The DNA damage response of irradiated V79 cells. V79 cells were exposed to 10 Gy (*a*), 5 Gy (*b*) and 2 Gy (*c*), using MRT patterned 5 keV X-rays and stained for γH2AX 1 h after irradiation. The microbeam widths were 100 µm with a centre-to-centre spacing of 200 µm. Individual nucleus intensity was quantified by determining the relative fluorescence using a separate targeted single-cell irradiation (*d*). Intensities were quantified using GeneTools (Syngene, Cambridge, UK). Error bars are standard error of the mean of four cell nuclei for each dose. Bars indicate 100 µm.

We performed MRT at 2 Gy, 5 Gy and 10 Gy using different beam widths and beam spacings. We observed a linear response with dose for fluorescence indicative of increased DNA double-strand breaks at higher doses (Fig. 3 ▶). In a separate experiment, to quantify the dose response signal, we targeted individual nuclei with 0 Gy, 2 Gy, 5 Gy and 10 Gy and determined the relative fluorescence. A dose response was observed (Fig. 3*d*).

### 3.4. Irradiation of part of the nucleus shows very little chromatin movement at early time points

**Figure 4:**
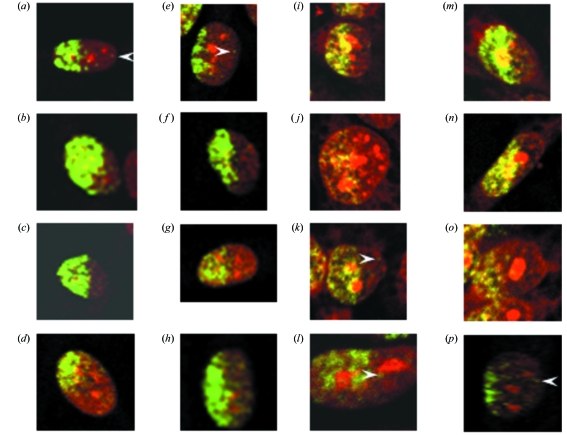
Partial cell irradiation with a 25 µm-wide beam of (*a*–*h*) N1-RGB cells and (*i*–*p*) V79 cells reveals clear demarcation of radiation-induced DNA double-strand breaks as determined by γH2AX foci. The in-air entrance dose was 10 Gy. Arrowheads point to γH2AX foci outside the apparent zone of irradiation. The brightness and contrast settings for some images (*c* and *l*) have been adjusted to compensate for staining variation. Some cell images are at a slightly higher zoom (*l*).

Only part of the nucleus was irradiated in cells at the edges of microbeam-targeted areas. This was evident at the 1 h time point but we were unable to draw this conclusion at the longer time point because the signal had decreased and dispersed by 12 h. There were very few γH2AX foci beyond the apparent radiation field edge at 1 h post-irradiation; however, in some cases γH2AX foci were observed near this area. These observations were consistent for both cell lines tested (Fig. 4 ▶).

## 4. Discussion

The 5.35 keV X-ray microbeam facility at the Photon Factory permits precise *in vitro* studies in sub-cellular radiobiology. One has the ability to emulate planar MRT using a slit system to make arbitrary beam widths and spacings. We were able to easily identify which cells had been irradiated by the microbeam using the γH2AX immunohistochemical marker of DNA damage.

We found the DNA damage response to this 5.35 keV microbeam radiation increased from 2 Gy to 10 Gy (Fig. 3*d*). A substantial amount of DNA repair had occurred over 12 h; however, there were above-background foci present at this time point. There are a number of possible reasons for these foci being present. First, they could be residual difficult-to-repair DNA damage which are not yet repaired or have retained the phosphorylation of H2AX histone. Alternatively, they could be a secondary event caused by the bystander effect which has been shown to have high levels near this time point in some cell systems (Sedelnikova *et al.*, 2007 ▶; Sokolov *et al.*, 2005 ▶). Another feature of the 12 h post-irradiated samples was that the γH2AX foci were not limited to the region of irradiation. This would be consistent with the bystander effect but it is also possible that there was cell movement to these locations within 12 h after irradiation. Both these possibilities may contribute to the differential advantages observed between tumour and normal tissue response to MRT when compared with conventional radiotherapy.

We also irradiated parts of the nucleus and in some cases irradiated exactly half of the nucleus to try to understand intra-nuclear responses to radiation. We observed a clear demarcation where the radiation-induced DNA double-strand breaks occurred as determined by γH2AX foci in half of the nucleus (Fig. 4 ▶). After 1 h there appeared to be very little movement of the chromatin; however, in a few cases it was evident that foci had formed beyond the edge of the irradiated area (Fig. 4 ▶; arrowheads). One explanation is that some double-strand break regional movement (up to a few micrometres) occurred within the first 30 s to 1 h. Similar effects have been observed with α-particle irradiation (Aten *et al.*, 2004 ▶). Alternatively, there may have been some early signalling of reactive oxygen species to cause DNA double-strand breaks outside the region of the irradiation zone. This would be consistent with previous reports of very little chromosome movement at the site of double-strand breaks within the nucleus after charged particles and ultrasoft X-rays (Jakob *et al.*, 2009 ▶; Nelms *et al.*, 1998 ▶; Soutoglou *et al.*, 2007 ▶; Splinter *et al.*, 2010 ▶; Tobias *et al.*, 2010 ▶).

The single-slit microbeam system allows us to investigate the effects of directly hit cells with less influence of confounding factors such as valley doses between the beams, although these factors may also play an important role in the response of tissue to MRT.

It is important to note that the dose between the 5.35 keV microbeams in this current study is virtually 0 Gy. The average range of secondary electrons in water produced by a 5.35 keV photon is very low, of the order of 1 µm based on stopping power theory and the CSDA (continuous slowing down approximation) theory. In contrast, the mean range of secondary electrons from a 100 keV X-ray beam is approximately 70 µm, which accounts for the valley dose in ‘conventional’ MRT using poly-energetic X-rays with a mean energy of about 100 keV. Photoelectric absorption is the dominant interaction mechanism in the 5 keV energy range and the probability of Compton scattering is low. For example, the total mass energy absorption coefficients (μ_en_/ρ) for water at 5 keV and 100 keV are 4.129 m^2^ kg^−1^ and 0.017 m^2^ kg^−1^, respectively (Khan, 2003 ▶). The total mass attenuation coefficient at 5 keV is dominated by the mass photoelectric attenuation coefficient (τ/ρ). This means that the dose-depositing electrons from the 5.35 keV X-ray beam deposit their energy close to the site of the incident photon. In addition, the effect of Fresnel diffraction by the slit edge is so small that the shape (square) of the uncovered area by the slit system is the same as the beam size at the sample position.

Therefore, the valley dose between successive peaks is essentially zero, using 5.35 keV X-rays. However, the valley dose is non-zero for poly-energetic MRT (50–250 keV) owing to Compton scattering of secondary electrons and, depending on the peak dose, may be several Gray. Nevertheless, we can exploit a 0 Gy valley dose with 5.35 keV microbeams to tease out radiobiological phenomena such as bystander effects, cellular communication and cell migration.

In conclusion, these *in vitro* microbeam studies carried out on the Photon Factory’s cellular radiobiology beamline may contribute to our understanding of the mechanisms of *in vivo* MRT which in turn could alter our understanding of fundamental radiation biology and therapy for cancer patients.

## References

[bb1] Aten, J. A., Stap, J., Krawczyk, P. M., van Oven, C. H., Hoebe, R. A., Essers, J. & Kanaar, R. (2004). *Science*, **303**, 92–95. 10.1126/science.1088845 14704429

[bb2] Azzam, E. I., de Toledo, S. M., Gooding, T. & Little, J. B. (1998). *Radiat. Res.* **150**, 497–504. 9806590

[bb3] Azzam, E. I., de Toledo, S. M., Spitz, D. R. & Little, J. B. (2002). *Cancer Res.* **62**, 5436–5442. 12359750

[bb4] Belyakov, O. V., Malcolmson, A. M., Folkard, M., Prise, K. M. & Michael, B. D. (2001). *Br. J. Cancer*, **84**, 674–679. 10.1054/bjoc.2000.1665 PMC2363796 11237389

[bb5] Bentzen, S. M. (2006). *Nat. Rev. Cancer*, **6**, 702–713. 10.1038/nrc1950 16929324

[bb6] Crosbie, J. C., Anderson, R. L., Rothkamm, K., Restall, C. M., Cann, L., Ruwanpura, S., Meachem, S., Yagi, N., Svalbe, I., Lewis, R. A., Williams, B. R. & Rogers, P. A. (2010). *Int. J. Radiat. Oncol. Biol. Phys.* **77**, 886–894. 10.1016/j.ijrobp.2010.01.035 20510199

[bb7] Delaney, G., Jacob, S., Featherstone, C. & Barton, M. (2005). *Cancer*, **104**, 1129–1137. 10.1002/cncr.21324 16080176

[bb8] Dilmanian, F. A., Button, T. M., Le Duc, G., Zhong, N., Pena, L. A., Smith, J. A., Martinez, S. R., Bacarian, T., Tammam, J., Ren, B., Farmer, P. M., Kalef-Ezra, J., Micca, P. L., Nawrocky, M. M., Niederer, J. A., Recksiek, F. P., Fuchs, A. & Rosen, E. M. (2002). *Neuro-Oncology*, **4**, 26–38. 10.1215/15228517-4-1-26 PMC1920629 11772430

[bb9] Dilmanian, F. A., Morris, G. M., Zhong, N., Bacarian, T., Hainfeld, J. F., Kalef-Ezra, J., Brewington, L. J., Tammam, J. & Rosen, E. M. (2003). *Radiat. Res.* **159**, 632–641. 10.1667/0033-7587(2003)159[0632:mechte]2.0.co;2 12710874

[bb10] Dilmanian, F. A., Qu, Y., Liu, S., Cool, C. D., Gilbert, J., Hainfeld, J. F., Kruse, C. A., Laterra, J., Lenihan, D., Nawrocky, M. M., Pappas, G., Sze, C. I., Yuasa, T., Zhong, N., Zhong, Z. & McDonald, J. W. (2005). *Nucl. Instrum. Methods Phys. Res. A*, **548**, 30–37. 10.1016/j.nima.2005.03.062 PMC1828126 17369874

[bb11] Gerashchenko, B. I. & Howell, R. W. (2003). *Cytometry A*, **56**, 71–80. 10.1002/cyto.a.10092 14608634

[bb12] Greening, J. R. (1985). *Fundamentals of Radiation Dosimetry*, 2nd ed. New York: Taylor and Francis.

[bb13] Han, W., Wu, L., Hu, B., Zhang, L., Chen, S., Bao, L., Zhao, Y., Xu, A. & Yu, Z. (2007). *Br. J. Radiol.* **80**, S7–S12. 10.1259/bjr/44550200 17704329

[bb14] Hei, T. K., Zhou, H., Ivanov, V. N., Hong, M., Lieberman, H. B., Brenner, D. J., Amundson, S. A. & Geard, C. R. (2008). *J. Pharm. Pharmacol.* **60**, 943–950. 10.1211/jpp.60.8.0001 PMC4410683 18644187

[bb15] Hickman, A. W., Jaramillo, R. J., Lechner, J. F. & Johnson, N. F. (1994). *Cancer Res.* **54**, 5797–5800. 7954402

[bb16] Iyer, R., Lehnert, B. E. & Svensson, R. (2000). *Cancer Res.* **60**, 1290–1298. 10728689

[bb17] Jakob, B., Splinter, J., Durante, M. & Taucher-Scholz, G. (2009). *Proc. Natl Acad. Sci. USA*, **106**, 3172–3177. 10.1073/pnas.0810987106 PMC2642473 19221031

[bb18] Kadhim, M. A., Macdonald, D. A., Goodhead, D. T., Lorimore, S. A., Marsden, S. J. & Wright, E. G. (1992). *Nature (London)*, **355**, 738–740. 10.1038/355738a0 1741061

[bb19] Kanai, T., Furusawa, Y., Fukutsu, K., Itsukaichi, H., Eguchi-Kasai, K. & Ohara, H. (1997). *Radiat. Res.* **147**, 78–85. 8989373

[bb20] Kashino, G., Kondoh, T., Nariyama, N., Umetani, K., Ohigashi, T., Shinohara, K., Kurihara, A., Fukumoto, M., Tanaka, H., Maruhashi, A., Suzuki, M., Kinashi, Y., Liu, Y., Masunaga, S., Watanabe, M. & Ono, K. (2009). *Int. J. Radiat. Oncol. Biol. Phys.* **74**, 229–236. 10.1016/j.ijrobp.2008.09.060 19362241

[bb21] Khan, F. (2003). *The Physics of Radiation Therapy*, 3rd ed. Baltimore: Williams and Wilkins.

[bb22] Kobayashi, K., Usami, N., Hieda, K., Takakura, K., Maezawa, H. & Hayashi, T. (2001). *Nucl. Instrum. Methods Phys. Res. A*, **467**–**468**, 1329–1332.

[bb23] Laissue, J. A., Blattmann, H., Wagner, H. P., Grotzer, M. A. & Slatkin, D. N. (2007). *Dev. Med. Child Neurol.* **49**, 577–581. 10.1111/j.1469-8749.2007.00577.x 17635201

[bb24] Laissue, J. A., Geiser, G., Spanne, P. O., Dilmanian, F. A., Gebbers, J. O., Geiser, M., Wu, X. Y., Makar, M. S., Micca, P. L., Nawrocky, M. M., Joel, D. D. & Slatkin, D. N. (1998). *Int. J. Cancer*, **78**, 654–660. 10.1002/(sici)1097-0215(19981123)78:5<654::aid-ijc21>3.0.co;2-l 9808538

[bb25] Lewis, D. A., Mayhugh, B. M., Qin, Y., Trott, K. & Mendonca, M. S. (2001). *Radiat. Res.* **156**, 251–258. 10.1667/0033-7587(2001)156[0251:poddan]2.0.co;2 11500134

[bb26] Littlefield, L. G., Hollowell, J. G. & Pool, W. H. (1969). *Radiology*, **93**, 879–886. 10.1148/93.4.879 5824247

[bb27] Lyng, F. M., Seymour, C. B. & Mothersill, C. (2000). *Br. J. Cancer*, **83**, 1223–1230. 10.1054/bjoc.2000.1433 PMC2363596 11027437

[bb28] Maeda, M., Usami, N. & Kobayashi, K. (2008). *J. Radiat. Res.* **49**, 171–180. 10.1269/jrr.07093 18187936

[bb29] Miura, M., Blattmann, H., Bräuer-Krisch, E., Bravin, A., Hanson, A. L., Nawrocky, M. M., Micca, P. L., Slatkin, D. N. & Laissue, J. A. (2006). *Br. J. Radiol.* **79**, 71–75. 10.1259/bjr/50464795 16421408

[bb30] Mothersill, C. & Seymour, C. (1997). *Int. J. Radiat. Biol.* **71**, 421–427. 10.1080/095530097144030 9154145

[bb31] Nagasawa, H. & Little, J. B. (1992). *Cancer Res.* **52**, 6394–6396. 1423287

[bb32] Narayanan, P. K., Goodwin, E. H. & Lehnert, B. E. (1997). *Cancer Res.* **57**, 3963–3971. 9307280

[bb33] Nelms, B. E., Maser, R. S., MacKay, J. F., Lagally, M. G. & Petrini, J. H. (1998). *Science*, **280**, 590–592. 10.1126/science.280.5363.590 9554850

[bb34] Prise, K. M. & O’Sullivan, J. M. (2009). *Nat. Rev. Cancer*, **9**, 351–360. 10.1038/nrc2603 PMC2855954 19377507

[bb35] Regnard, P., Brauer-Krisch, E., Tropres, I., Keyriainen, J., Bravin, A. & Duc, G. L. (2008). *Eur. J. Radiol.* **68**, S151–S155. 10.1016/j.ejrad.2008.04.049 18635331

[bb36] Regnard, P., Le Duc, G., Bräuer-Krisch, E., Troprès, I., Siegbahn, E. A., Kusak, A., Clair, C., Bernard, H., Dallery, D., Laissue, J. A. & Bravin, A. (2008). *Phys. Med. Biol.* **53**, 861–878. 10.1088/0031-9155/53/4/003 18263945

[bb37] Sedelnikova, O. A., Nakamura, A., Kovalchuk, O., Koturbash, I., Mitchell, S. A., Marino, S. A., Brenner, D. J. & Bonner, W. M. (2007). *Cancer Res.* **67**, 4295–4302. 10.1158/0008-5472.CAN-06-4442 17483342

[bb38] Sedelnikova, O. A., Rogakou, E. P., Panyutin, I. G. & Bonner, W. M. (2002). *Radiat. Res.* **158**, 486–492. 10.1667/0033-7587(2002)158[0486:qdoiid]2.0.co;2 12236816

[bb39] Serduc, R., van de Looij, Y., Francony, G., Verdonck, O., van der Sanden, B., Laissue, J., Farion, R., Bräuer-Krisch, E., Siegbahn, E. A., Bravin, A., Prezado, Y., Segebarth, C., Rémy, C. & Lahrech, H. (2008). *Phys. Med. Biol.* **53**, 1153–1166. 10.1088/0031-9155/53/5/001 18296755

[bb41] Seymour, C. & Mothersill, C. (2006). *Dose Response*, **4**, 277–282. 10.2203/dose-response.06-116.Seymour PMC2477676 18648585

[bb40] Seymour, C. B. & Mothersill, C. (1997). *Radiat. Oncol. Investig.* **5**, 106–110. 10.1002/(SICI)1520-6823(1997)5:3<106::AID-ROI4>3.0.CO;2-1 9303065

[bb42] Smilowitz, H. M., Blattmann, H., Bräuer-Krisch, E., Bravin, A., Di Michiel, M., Gebbers, J. O., Hanson, A. L., Lyubimova, N., Slatkin, D. N., Stepanek, J. & Laissue, J. A. (2006). *J. Neurooncol.* **78**, 135–143. 10.1007/s11060-005-9094-9 16598429

[bb43] Sokolov, M. V., Smilenov, L. B., Hall, E. J., Panyutin, I. G., Bonner, W. M. & Sedelnikova, O. A. (2005). *Oncogene*, **24**, 7257–7265. 10.1038/sj.onc.1208886 16170376

[bb44] Soutoglou, E., Dorn, J. F., Sengupta, K., Jasin, M., Nussenzweig, A., Ried, T., Danuser, G. & Misteli, T. (2007). *Nat. Cell Biol.* **9**, 675–682. 10.1038/ncb1591 PMC2442898 17486118

[bb45] Splinter, J., Jakob, B., Lang, M., Yano, K., Engelhardt, J., Hell, S. W., Chen, D. J., Durante, M. & Taucher-Scholz, G. (2010). *Mutagenesis*, **25**, 289–297. 10.1093/mutage/geq005 PMC2902920 20167590

[bb46] Tobias, F., Durante, M., Taucher-Scholz, G. & Jakob, B. (2010). *Mutat. Res.* **704**, 54–60. 10.1016/j.mrrev.2009.11.004 19944777

[bb47] Tomita, M., Maeda, M., Maezawa, H., Usami, N. & Kobayashi, K. (2010). *Radiat. Res.* **173**, 380–385. 10.1667/RR1995.1 20199223

[bb48] Usami, N., Maeda, M., Eguchi-Kasai, K., Maezawa, H. & Kobayashi, K. (2006). *Radiat. Prot. Dosim.* **122**, 307–309. 10.1093/rpd/ncl434 17182605

[bb49] Yang, H., Asaad, N. & Held, K. D. (2005). *Oncogene*, **24**, 2096–2103. 10.1038/sj.onc.1208439 15688009

